# A Novel Modeling in Mathematical Biology for Classification of Signal Peptides

**DOI:** 10.1038/s41598-018-19491-y

**Published:** 2018-01-18

**Authors:** Asma Ehsan, Khalid Mahmood, Yaser Daanial Khan, Sher Afzal Khan, Kuo-Chen Chou

**Affiliations:** 1 University of the Punjab, Department of Mathematics, Lahore, 54500 Pakistan; 2 University of Management and Technology, School of Systems and Technology, Department of Computer Science, Lahore, 54770 Pakistan; 3 King Abdul Aziz University, Faculty of Computing and Information Technology in Rabigh, Jeddah, 21577 Saudi Arabia; 4 Gordon Life Science Institute, San Diego, CA 92130 USA; 5 Abdul Wali Khan University, Department of Computer Sciences, Mardan, Pakistan

## Abstract

The molecular structure of macromolecules in living cells is ambiguous unless we classify them in a scientific manner. Signal peptides are of vital importance in determining the behavior of newly formed proteins towards their destined path in cellular and extracellular location in both eukaryotes and prokaryotes. In the present research work, a novel method is offered to foreknow the behavior of signal peptides and determine their cleavage site. The proposed model employs neural networks using isolated sets of prokaryote and eukaryote primary sequences. Protein sequences are classified as secretory or non-secretory in order to investigate secretory proteins and their signal peptides. In comparison with the previous prediction tools, the proposed algorithm is more rigorous, well-organized, significantly appropriate and highly accurate for the examination of signal peptides even in extensive collection of protein sequences.

## Introduction

**Figure 1:**
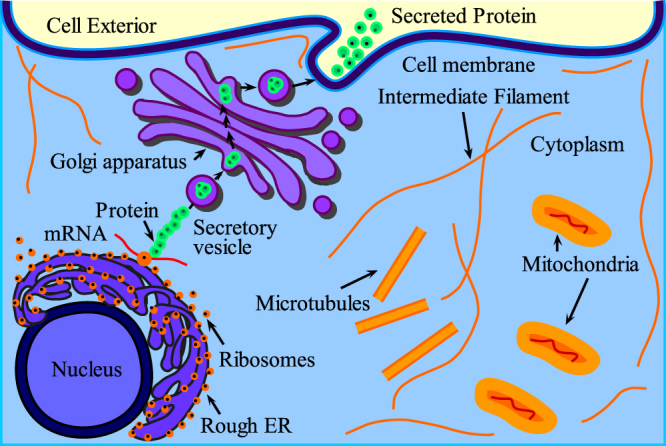
Protein secretion: Ribosomes deposits the protein in endoplasmic reticulum (ER), protein exits ER and enters Golgi apparatus for processing and later it exits Golgi and enters into the cell exterior.

The classification of proteins bears vast interest to researchers across the world. For better understanding of the molecular structure of living cells, it is important to classify and categorize their macromolecules like proteins^1^ in terms of their attributes. To examine the behavior of newly synthesized proteins towards cellular and extracellular positions in cells (for both eukaryotes and prokaryotes), signal peptide works like a “ZIP code”^2,3^. The study of signal can help to propose new medications for genetic remedial treatment which has become difficult for pharmaceutical chemists to develop more accurately^4,5^. A newly translated secretory protein starts to move from rough endoplasmic reticulum to Golgi transport vesicles and then Golgi cisternae leading to secretory transport vesicles. Consequently, these are secreted to the cell exterior surface. The secretory proteins enter the exterior surface of a cell via protein conducting channels (see Fig. 1). The signal peptides translocate the newly created proteins in the secretory pathway^6,7^.

It is worth mentioning that the signal peptide of newly synthesized protein can diverge under abnormal circumstances from the exact path. This deviation results the protein ending up in an inappropriate cellular location and hence causes severe diseases. Hereafter, an accurate model for prediction of signal peptide is of much more crucial importance. Predictive results are momentous to examine cell functions and analyze its prospective and genetic purposes^8^. For the prediction of arbitrary signal sequences, a capable novel modeling with a consolidated approach is to be devised to provide more assiduous results. Everyday, a huge number of protein sequences are collected and entering into databanks. Indeed, it is extremely desirable to develop a robust, reliable and excellently accurate computational method for the prediction of signal sequences. A number of researchers presented several models in this respect^9–16^. A huge amount of literature is found pertaining to such prediction models^3,4,17–20^. PrediSi^21^ associated the prediction techniques for secretory and non-secretory protein and proposed position weight matrix (PWM) approach for identification of cleavage site. SignalP^22^ developed a method that uses a hidden Markov model (HMM) while Signal-CF^8^ suggested a coupling fusion predictor that contrived through comprising the subsite coupling effects on protein sequences. Gunnar Von Heijne developed a prototype model depicting the nearby amino acids signal sequences^23^. Lal *et al*. devised a scheme for identifying proteins containing signal peptides and assigned a label SP to a protein after knowing that a protein contains a signal^24^. Extreme learning machine (ELM) and improved ELM were also devised to categorize the avalanche of protein sequences^25,26^. Some researcher have worked on identifying protein sub-cellular localization using “Support Vector Machine” (SVM) by targeting amino acid composition^27,28^. The SVMs and other machine learning classifiers (ANN, random forest) have been widely used in the field of bioinformatics, and some predictors have been established based on these classifiers, such as PSFM-DBT^29^, 2L-piRNA^30^, Pse-Analysis^31^, ProtDec-LTR^32^, ProtDec-LTR2.0^33^, etc. Some powerful protein analysis methods have been proposed for the formulation of biological sequences, such as Pse-in-One^31^, repDNA^34^, based on different functions to produce feature vector for biological sequences. HMMTOP was developed for the prediction of localization of helical transmembrane^35^. Kuo-Chen Chou devised quasi-sequence-order effect for the prediction of sub-cellular localization^36^ and afterwards classified protein sequences employing Pseudo Amino Acid Composition^37^. Multiple classifications of protein are offered in^38^. The perception of difference between signal sequence and its peptide chain is manipulated by predictors SS- and SP-indexes^39^. SPOCTOPUS: a combined predictor of signal peptides and membrane protein topology based on neural networks, hidden Markov model (HMM) and dynamic programming algorithm is proposed in^40^. Various researcher have used different mathematical and statistical models for solving various classification problems^41–43^. Yaser *et al*.^44,45^ proposed another methodological rigorous examination for the prediction of membrane protein.

## Results

In order to validate the current model, a comparative analysis with existing models^8,14,21,22^ is established. It is found that the techniques suggested in PrediSi^21^, Neural Network (NN)^14^ and hidden Markov model (HMM)^22^ were less efficient than the Signal-CF^8^. Although the PredSi denied schemes such as NN and HMM^14,22^. Moreover, Signal-CF is a predictor established using pseudo amino acid composition, scaled window and subsite coupling effect, and is an improvement over the PredSi and SignalP methods. Also, few disadvantages of the previous predictors were observed. For example, the feature vector in Chou’s scheme was dependent on a the value of a variable *λ*.

As for the current framework, the occurrence of each residue with a special weight factor has been incorporated for all protein sequences. This yielded a comprehensive description for any arbitrary sequence. A better accuracy rate has been achieved as compared to previous models. Self-consistency test, 10-fold cross validation and jackknife testing were conducted to ensure the accuracy of the proposed model. These tests are elaborated in Tables 1 and 2. Table 1 provides accuracy outcomes for the prediction of secretory protein of eukaryotic, Gram-positive and Gram-negative datasets whereas Table 2 represents the same statistical analysis for non-secretory proteins.

**Table 1:** Prediction accuracies for secretory protein for prokaryotes (Gram-positive & Gram-negative) and eukaryotes.

Datasets	Self-consistency Test	Cross validation	Jackknife Test
Eukaryotic(%)	95.00	93.03	92.98
Gram-positive(%)	93.50	87.90	87.85
Gram-negative(%)	97.70	97.09	97.04

**Table 2:** Prediction accuracies of non-secretory protein for prokaryotes (Gram-positive & Gram-negative) and eukaryotes.

Datasets	Self-consistency Test	Cross validation	Jackknife Test
Eukaryotic(%)	94.60	92.35	92.30
Gram-positive(%)	91.50	86.55	86.50
Gram-negative(%)	95.80	94.37	94.32

In previous works, most of the methodologies were evaluated by self-consistency, cross validation or/and jackknife testing. The proposed model is validated by all the three testing procedures simultaneously. Firstly, self-consistency test is performed. This is applied to the trained neural network predictor. Datasets for eukaryotic, Gram-positive and Gram-negative are separated into subsets of positives and negatives with comparable random sizes. After the completion of the training process, the test is executed. In further steps, the other two validation techniques, namely cross validation and jackknife are also evaluated.

**Figure 2:**
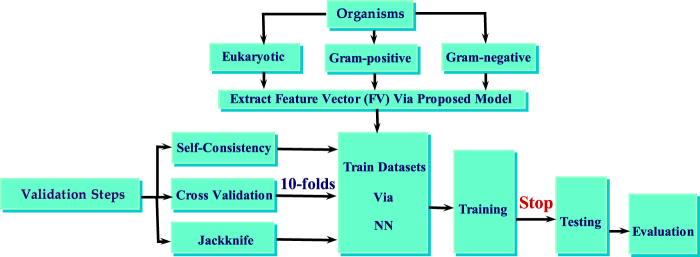
Flowchart illustrating the procedure of training, testing and evaluation.

**Figure 3:**
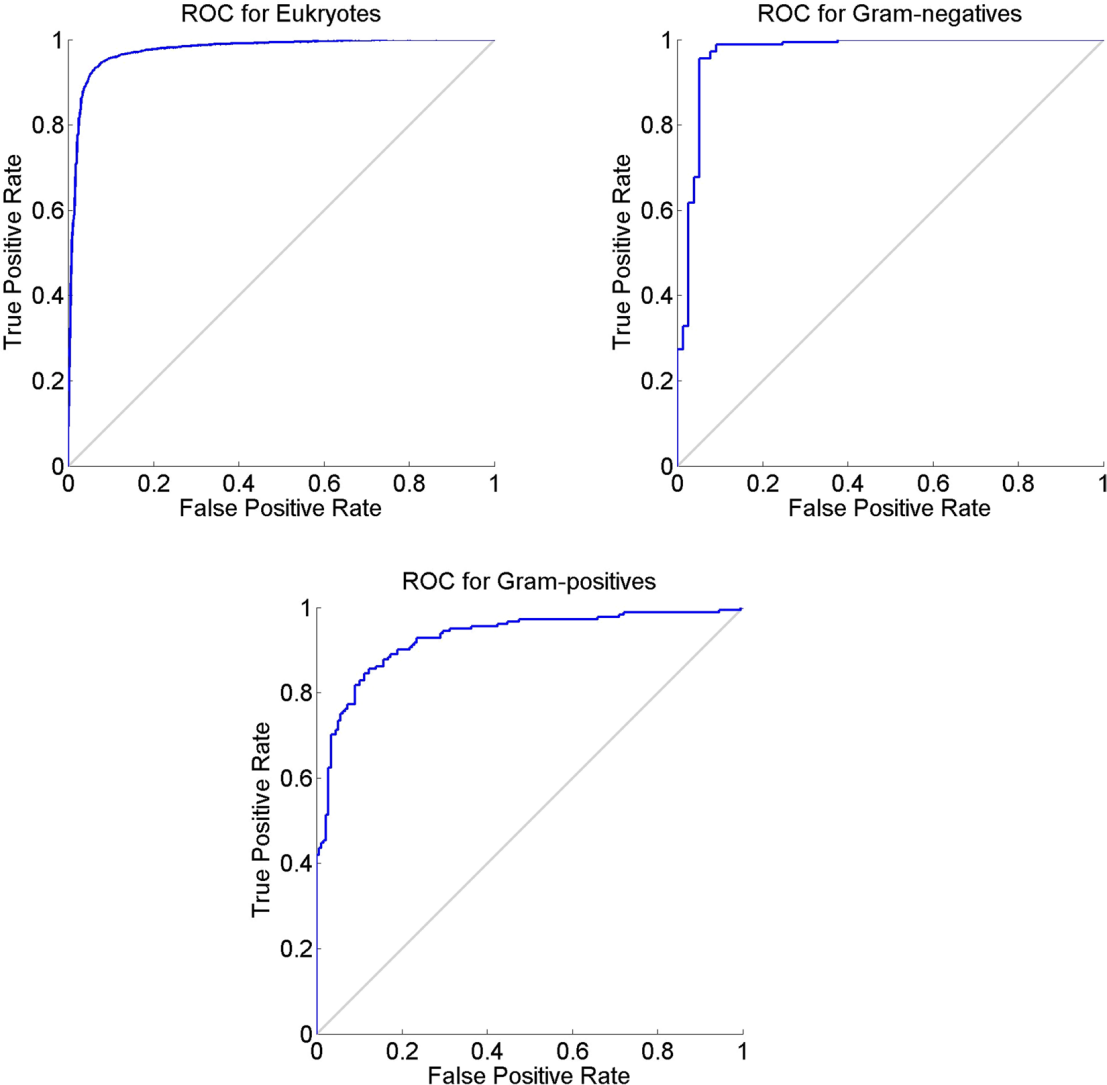
ROC of Self-consistency test for Eukaryotes, Gram Negatives and Gram Positives.

The validation process is demonstrated in the following flow chart (see Fig. 2). Secretory and non-secretory datasets are divided into ten equal units for 10-fold cross validation. Evaluation is performed based on performance in ten dissimilar and disjoint subsets of test data and is translated into ten distinctive confusion matrices together with the ROC (Receiver operating characteristic) graphs. The overall prediction accuracy is achieved by averaging all the outcomes. The prediction accuracies are estimated for each subset separately. This procedure is necessary for cross validation and jackknife testing as well. The ROC is a graphical interpretation for the performance of a classifier which illustrates the false positive rate (FPR) against the true positive rate (TPR). The area under the curve signifies the accuracy of the system. ROC graph yielded as a result of self consistency test for the three organisms dataset in given below (see Fig. 3).

A comparative analysis of the proposed model with existing techniques is elaborated in Table 3. It is found that the previous accuracy rates varied from 72% to 87% whereas the accuracy of the proposed model for Self-consistency test lies between 95% to 97%.

**Table 3:** A comparison of proposed model for **Self-consistency** test in order to predict secretory and non-secretory protein sequences.

Protein’s Sort	dataset	PridiSi	NN(SignalP)	HMM(SignalP)	Signal-CF	Proposed Model
Secretory	Eukaryotic	72.66	82.11	78.73	76.50	**95**.**00**
	Gram-positive	78.39	77.97	75.42	80.50	**93**.**50**
	Gram-negative	86.54	86.54	87.07	87.80	**97**.**70**
Non-secretory	Eukaryotic	98.31	99.21	97.74	99.78	**94**.**60**
	Gram-positive	97.89	93.25	99.16	99.79	**91**.**50**
	Gram-negative	95.70	96.24	99.10	99.82	**95**.**80**

In order to assess the accuracy of the prediction model four distinct metrics were utilized. The expected success rate and performance of the proposed model is predicted by these statistical measures. To understand these statistical metrics in easiest way, the prediction scales are expanded as discussed in^46,47^. The true prediction rates for secretory ${\Upsilon }^{+}$ and non-secretory ${\Upsilon }^{-}$ for three describes organisms categories are given by

1 $${\Upsilon }^{+}=\frac{{{\mathbb{T}}}^{+}-{{\mathbb{T}}}_{-}^{+}}{{{\mathbb{T}}}^{+}}$$

2 $${\Upsilon }^{-}=\frac{{{\mathbb{T}}}^{-}-{{\mathbb{T}}}_{+}^{-}}{{{\mathbb{T}}}^{-}}$$

where ${{\mathbb{T}}}^{+}$ and ${{\mathbb{T}}}_{-}^{+}$ represents the total number of the predicted secretory proteins and incorrectly identified non-secretory protein sequences. Similarly ${{\mathbb{T}}}^{-}$ and ${{\mathbb{T}}}_{+}^{-}$ denotes the total number of anticipated non-secretory peptides and falsely recognized secretory protein chains. In general the prediction rate is defined by

3 $$\Upsilon =\frac{{\Upsilon }^{+}{{\mathbb{T}}}^{+}+{\Upsilon }^{-}\,{{\mathbb{T}}}^{-}}{{{\mathbb{T}}}^{+}+{{\mathbb{T}}}^{-}}=1-\frac{{{\mathbb{T}}}_{-}^{+}+{{\mathbb{T}}}_{+}^{-}}{{{\mathbb{T}}}^{+}+{{\mathbb{T}}}^{-}}$$

From the equations (1) to (3), it is clear that in case there is no incorrectly predicted secretory and non-secretory protein sequences i.e., ${{\mathbb{T}}}_{-}^{+}={{\mathbb{T}}}_{+}^{-}=0$ then, ${\Upsilon }^{+}={\Upsilon }^{-}=1$ and the complete prediction accuracy rate is $\Upsilon =1$. Conversely, when ${{\mathbb{T}}}_{-}^{+}={{\mathbb{T}}}_{+}^{-}\ne 0$ then the prediction rate would be lesser than 1.

Furthermore, it is helpful to emphasize the importance of equation (4) which is frequently found in literatures for observing the performance superiority of a predictor. Particularly, its advantages have been analyzed and endorsed by a series of significant studies published very recently^48–53^.

4 $$(\begin{array}{rcl}Sn & = & \frac{TP}{TP+FN}\\ Sp & = & \frac{TN}{TN+FP}\\ Acc & = & \frac{TP+TN}{TP+TN+FP+FN}\\ MCC & = & \frac{TP\times TN-FP\times FN}{\sqrt{(TP+FP)\,(FN+TN)\,(FP+TN)\,(TP+FN)}}\end{array}$$

where TP, TN, FP and FN represents the true positive, true negative, false positive and false negative values respectively while Sn, Sp, Acc and MCC denotes the values for sensitivity, specificity, accuracy and Mathew’s correlation coefficient.

The equation (4) can be rewritten in the form of the symbols given in (3)

5 $$(\begin{array}{rcl}TP & = & {{\mathbb{T}}}^{+}-{{\mathbb{T}}}_{-}^{+}\\ TN & = & {{\mathbb{T}}}^{-}-{{\mathbb{T}}}_{+}^{-}\\ FP & = & {{\mathbb{T}}}_{+}^{-}\\ FN & = & {{\mathbb{T}}}_{-}^{+}\end{array}$$

By substituting (5) into (4) along and utilizing (3), we have

6 $$(\begin{array}{rcl}Sn & = & 1-\frac{{{\mathbb{T}}}_{-}^{+}}{{{\mathbb{T}}}^{+}}\\ Sp & = & 1-\frac{{{\mathbb{T}}}_{+}^{-}}{{{\mathbb{T}}}^{-}}\\ Acc & = & \Upsilon =1-\frac{{{\mathbb{T}}}_{-}^{+}+{{\mathbb{T}}}_{+}^{-}}{{{\mathbb{T}}}^{+}+{{\mathbb{T}}}^{-}}\\ MCC & = & \frac{1-(\frac{{{\mathbb{T}}}_{-}^{+}}{{{\mathbb{T}}}^{+}}+\frac{{{\mathbb{T}}}_{+}^{-}}{{{\mathbb{T}}}^{-}})}{\sqrt{(1+\frac{{{\mathbb{T}}}_{+}^{-}-{{\mathbb{T}}}_{-}^{+}}{{{\mathbb{T}}}^{+}})\,(1+\frac{{{\mathbb{T}}}_{-}^{+}-{{\mathbb{T}}}_{+}^{-}}{{{\mathbb{T}}}^{-}})}}\end{array}$$

It is worth mentioning that from (6) that if ${{\mathbb{T}}}_{-}^{+}=0$ then *Sn* = 1 signifying that no secretory protein sequences is incorrectly predicted as non-secretory protein sequence. In other case when ${{\mathbb{T}}}_{-}^{+}={{\mathbb{T}}}^{+}$ implies that *Sn* = 0 represents that all the secretory protein sequences were incorrectly predicted as non-secretory protein chains. Similarly if ${{\mathbb{T}}}_{+}^{-}=0$ yields *Sp* = 1 then it signifies that no non-secretory sequence was incorrectly predicted similarly if ${{\mathbb{T}}}_{+}^{-}={{\mathbb{T}}}^{-}$ gives *Sp* = 0 then it shows that all the non-secretory sequences were falsely predicted as secretory sequences. On the other hand the prediction accuracy metric $Acc=\Upsilon =1$ when there is no incorrectly predicted sequences for secretory ${\Upsilon }^{+}$ as well as for non-secretory proteins ${\Upsilon }^{-}$ i.e., ${{\mathbb{T}}}_{-}^{+}={{\mathbb{T}}}_{+}^{-}=0$. A value of ${{\mathbb{T}}}_{-}^{+}={{\mathbb{T}}}^{+}$ and ${{\mathbb{T}}}_{+}^{-}={{\mathbb{T}}}^{-}$ indicates that all the secretory ${\Upsilon }^{+}$ and non-secretory ${\Upsilon }^{-}$ sequences were falsely predicted hence yielding an overall accuracy $Acc=\Upsilon =0$. Additionally, Matthew correlation coefficient (MCC) is frequently used to assess the performance of binary classifications. MCC is designed in such a way that the disparity in the size of positive or negative samples in the comprehensive dataset does not bias the overall outcome.

The statistical analysis using independent set testing for computing these metrics mentioned above is given in Table 4 with a noteworthy MCC value for the three segregated Eukrayotic, Gram-positive and Gram-negative datasets is 0.81, 0.71 and 0.90 respectively.

**Table 4:** Depicts the validation metrics for the proposed model.

Datasets	TP	TN	FP	FN	Acc	Mcc	Sn	Sp
Eukaryotic	5648	1297	392	92	0.94	0.81	0.98	0.77
Gram-positive	159	148	22	31	0.85	0.71	0.84	0.87
Gram-negative	177	71	6	6	0.95	0.90	0.97	0.92

## Discussion

Undoubtedly, the examination and analysis of patterns and sequence is quite convoluted when there are avalanche of protein sequences with diverse lengths. The statistical formulation of these sequences along with construction of robust data set to produce assiduous results was a challenging task. These cumbersome tasks have been rectified by the proposed technique. A performance comparison over the existing and previous predictors^8,14,21,22^ has been analyzed and a worth seeing prediction accuracy using the proposed technique was observed. The idea was to recognize the query proteins as secretory or non-secretory and hence identify the presence of signal peptides, further in the next step the cleavage site for the signal peptide is identified based on “(−3, −1) -Rule”, as described by Von Heijni^10^. It is obseved that signal peptides are more important than protein synthesization. The insufficiency of protein in distribution or contribution is cause of health hazards. So, the protein synthesization is not enough to perform cell functions properly but the compartmentalization of proteins to their relevent loci is of vital importance. Involvement of deviated protein with beneficial cells function is responsible for severe health diseases including Neurodegenerative disorder and cells death^54,55^. Hence, the computational capability to classify a protein as secretory or a non-secretory protein with a high level of accuracy bears great significance. In this process of anticipation, a large-scaled benchmark data set had been selected and incorporated into the prediction model. Even for huge data set, signal sequences along with their cleavage site was predicted at the cost of minor computational overhead with a high accuracy. The proposed algorithm can also be applied in future works to solve several other problems such as identification of Post Translational Modification (PTM) sites^56–58^, DNA-binding protein prediction^59^, protein-protein interaction prediction^60^, etc. The prediction accuracy of the proposed algorithm using self-consistency, cross validation and jackknife tests were reported as **95**.**00%**, **93**.**50%** and **97**.**70%** (Self-consistency), **93**.**03%**, **87**.**90%** and **97**.**90%** (Cross validation) and **92**.**98%**, **87**.**85%** and **97**.**04%** (Jackknife) for the three organisms namely eukaryotic, Gram-positive and Gram-negative respectively.

## Methods

### Material

**Figure 4:**
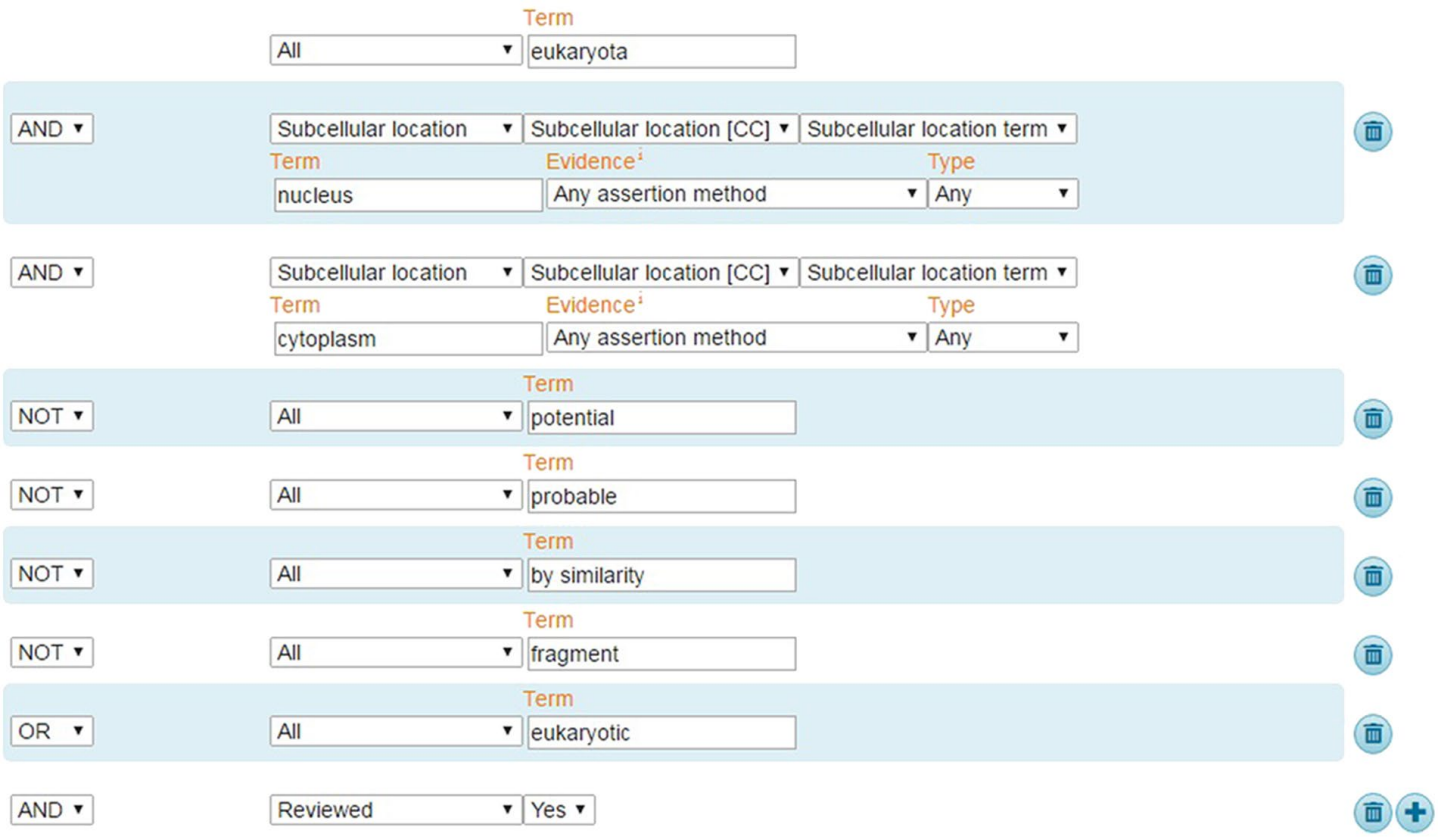
Shows a sample query to extract dataset.
- In taxonomy field, the keyword eukaryota was combed to obtain a training dataset for secretory protein sequences. The proteins annotated as signal peptides were only selected excluding those containing ambiguous keywords like potential, probable, fragment and by similarity in FT field. Out of those selected protein sequences, in which the preamble signal peptide was surely contained, the first 100 residues were extracted. The Gram-positive and Gram-negative organisms data was also extracted in a similar fashion.
- The defined steps were adopted strictly to collect a first-rate benchmark data set of signal peptides for both secretory and non-secretory proteins in the three organisms namely eukaryotic, Gram-positive and Gram-negative.
- Table 5, shows that 28,220 eukaryotic, 934 Gram-positive and 1046 Gram-negative samples were found in all 30,200 secretory proteins. After deleting similar entities, these are reduced to 25327 unique values. Similarly for all non-secretory proteins, 5595, 908 and 441 were found in eukaryotic, Gram-positive and Gram-negative respectively and 6,944 conjointly. After deleting repeated items, these are reduced to 6426 unique values. The benchmark dataset extracted for the proposed model is more diverse and comprehensive than the benchmark data set employed by existing predictors. Within the training dataset the secretory samples are considered as positive and non-secretory samples as negative as shown in flowchart given below (see Fig. 5).

Since user-friendly and publicly accessible web-servers represent the future direction for developing practically more useful models^61–63^, we shall make efforts in our future work to provide a web-server for the method presented in this paper. A current updated edition of the well-known database uniprot wasn employed to obtain an inclusive benchmark dataset. The proposed model was trained over the assimilated dataset and subsequently validated in the following steps:

- The eukaryotic or eukaryota, Gram-positive and Gram-negative are the organisms which have been incorporated for the desired prediction via proposed model. A benchmark data set related to these organisms was composed. A query was operated for the extraction of non-secretory protein dataset in the entire database. Through this inquiry, those protein sequences were selected which were marked in OC (Organism Classification) field as eukaryotic or eukaryota, Gram-positive or Gram-negative. Amongst these extracted entities, the eukaryotic entries annotated with subcellular locations as the nucleus or cytoplasm were selected while Gram-positive and Gram-negative entries annotated with subcellular location as cytoplasm were selected as a non-secretory protein sequences shown below (see Fig. 4). The vague extraction glossed with keywords like “by similarity” and “fragment” were discarded.

**Table 5:** The data set used to test the proposed model includes secretory and non-secretory protein sequences in all three organisms.

Organisms	Eukaryotic (K)	Gram-positive (*G*_+_)	Gram-negative (*G*_−_)
Secretory	28,220	934	1,046
Non-secretory	5,595	908	441
Total	33,815	1,842	1,487

**Figure 5:**
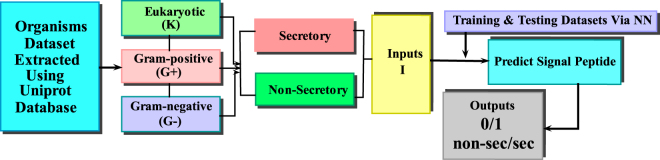
A flowchart depicting the process starting from data extraction and ultimately leading to validation of results.

In the above flow chart for the sake of convenience inputs expressed as positive (negative) in the direction of secretory (non-secretory) for eukaryotic (**K**), Gram-positive(*G*_+_) and Gram-negative(*G*_−_) are documented as follows.

$$\begin{array}{rcl}{I}_{K} & = & {K}^{+}\oplus {K}^{-}\\ {I}_{{G}_{+}} & = & {G}_{+}^{+}\oplus {G}_{+}^{-}\\ {I}_{{G}_{-}} & = & {G}_{-}^{+}\oplus {G}_{-}^{-}\end{array}$$

where *I*_*K*_, ${I}_{{G}_{+}}$ and ${I}_{{G}_{-}}$ are the inputs from eukaryotic, Gram-positive and Gram-negative organisms for the collection of secretory (*K*^+^, ${G}_{+}^{+}$, ${G}_{-}^{+}$) and non-secretory (*K*^−^, ${G}_{+}^{-}$, ${G}_{-}^{-}$) protein sequences. And the symbol $\oplus$ denotes the combination of both secretory and non-secretory protein sequences. The primary structure of secretory and non-secretory proteins can be found in Supplementary Tables S1 and S2 respectively.

### Method

Consider a protein sequence **S** comprising of *N* amino acid residues.

7 $${\bf{S}}={\xi }_{1}{\xi }_{2}{\xi }_{3}{\xi }_{4}{\xi }_{5}{\xi }_{6}{\xi }_{7}\cdots {\xi }_{N}$$

where *ξ*_1_, is the first amino acid residue, *ξ*_2_ is the second amino acid residue and so on up till the last residue *ξ*_*N*_ within the polypeptide chain **S**. Also, *N* represents the length of protein sequence (7). In order to predict the signal peptide in a convenient manner a computational algorithm has been proposed. This algorithm preserves the sequence order effect and is carried out by assimilating the whole sequence data together with the occurrence of each amino acid residue ${{\rm{\Lambda }}}_{\hat{a}}$ of type $\hat{a}\mathrm{:}1\le \hat{a}\le 20$ (any one of residue among twenty amino acid residues). The whole structure is based on expressions (8) to (11). Expression (8) is associated with the number of occurrence ${{\rm{\Lambda }}}_{\hat{a}}$ of residue $\hat{a}$ and with the possible number of correlated factors *ϑ* of $\hat{a}$ with itself such that $({{\rm{\Lambda }}}_{\hat{a}}-\mathrm{1)!}{\vartheta }({\xi }_{\hat{a}},{\xi }_{\hat{a}})$. Subsequently, expression (9) represents the mean factors *M*_0_, *M*_*i*_ and *M*_*j*_ which are linked with the difference factors of $\hat{a}$ at their corresponding positions and is appended with constraint (10). Moreover, *M*_*i*_ varies according to the number of difference factors and each difference factor associates an exclusive mean term. The difference is labeled as (*q* − *p*)_*i*_, where p and q are respective positions of $\hat{a}$ in the polypeptide chain given as ${\xi }_{p}={\xi }_{q}={\xi }_{\hat{a}}$. Also subscript *i* represents the number of the possible difference factors between similar residues excluding first and last time occurrence of residue, $\hat{a}$ depends upon the *n* number of $\hat{a}$ residues in sequence. Likewise *M*_*j*_ is fitted to factor (*N* − *r*), where *r* is the position of the *n*^*th*^ occurrence of the residue $\hat{a}$ in sequence (7) such that ${\xi }_{r}={\xi }_{\hat{a}}$ and 1 ≤ *p* < *q* < *r* ≤ *N* represents the $\hat{a}$ residue at their corresponding positions.

8 $${{\rm{\Lambda }}}_{\hat{a}}+({{\rm{\Lambda }}}_{\hat{a}}-\mathrm{1)!}\,{\vartheta }({\xi }_{\hat{a}},{\xi }_{\hat{a}})$$

9 $$\begin{array}{l}[{(p-\mathrm{0)}}_{0}{M}_{0}+\sum _{q > p}{(q-p)}_{i}{M}_{i}+{(N-r)}_{n}{M}_{j=n}];\\ 1\le p < q < r\le N,\,i=1,2,3,\ldots n-1\end{array}$$

10 $$\mathrm{(9)}\Rightarrow (\begin{array}{l}\sum _{q > p}{(q-p)}_{i}{M}_{i}+{(N-r)}_{n}{M}_{j=n},\,if\quad 1\le p < q,r < N\\ {(p-\mathrm{0)}}_{0}{M}_{0}+\sum _{q > p}{(q-p)}_{i}{M}_{i}+{(N-r)}_{n}{M}_{j=n},\,if\quad 1 < p < q,r < N\\ {(p-\mathrm{0)}}_{0}{M}_{0}+\sum _{q > p}{(q-p)}_{i}{M}_{i},\,if\quad 1 < p < q,r=N\end{array}$$

Combining expression (8) and (9) and using constraint (10) yields the template for manipulating feature component related to $\hat{a}$, given in (11) and (12).

11 $$\begin{array}{l}{{\rm{\Lambda }}}_{\hat{a}}+({{\rm{\Lambda }}}_{\hat{a}}-\mathrm{1)!}\,{\vartheta }({\xi }_{\hat{a}},{\xi }_{\hat{a}})+[{(p-\mathrm{0)}}_{0}{M}_{0}+\sum _{q > p}{(q-p)}_{i}{M}_{i}+{(N-r)}_{n}{M}_{j=n}],\\ \quad i=1,2,3,\ldots ,n-1\end{array}$$

Or

12 $$\begin{array}{c}{{\rm{\Lambda }}}_{\hat{a}}+({{\rm{\Lambda }}}_{\hat{a}}-\mathrm{1)!}\,{\vartheta }({\xi }_{\hat{a}},{\xi }_{\hat{a}})+[(p-{\mathrm{0)}}_{0}{M}_{0}+{(q-p)}_{1}{M}_{1}+{(q-p)}_{2}{M}_{2}\\ \quad \,\,\,+{(q-p)}_{3}{M}_{3}+\cdots +{(q-p)}_{n-1}{M}_{n-1}+{(N-r)}_{n}{M}_{j=n}]\end{array}$$

whereas *M*_*i*_ and *M*_*j*_ are count factors of residue $\hat{a}$ with other nineteen residues occurring before and after $\hat{a}$ respectively and can be defined in terms of *X* and *Y*, as elaborated in equations (13) and (14).

13 $$\begin{array}{rcl}{M}_{i} & = & \{{X}_{i}+{Y}_{i}\},\quad i=1,2,3,\ldots ,n-1\\ {M}_{j=n} & = & \{X+Y\}\end{array}$$

where

14 $$\begin{array}{rcl}X & = & {X}_{i}=\frac{1}{38}[\sum _{\begin{array}{l}k\mathrm{=1}\\ k\ne \hat{a}\end{array}}^{20}{f}_{k}{\vartheta }({\xi }_{k},{\xi }_{\hat{a}})+\sum _{\begin{array}{l}k\mathrm{=1}\\ k\ne \hat{a}\end{array}}^{20}{f}_{0}{\vartheta }({\xi }_{\hat{a}},{\xi }_{k})]\\ Y & = & {Y}_{i}=\frac{1}{38}[\sum _{\begin{array}{l}k\mathrm{=1}\\ k\ne \hat{a}\end{array}}^{20}{f}_{0}{\vartheta }({\xi }_{k},{\xi }_{\hat{a}})+\sum _{\begin{array}{l}k\mathrm{=1}\\ k\ne \hat{a}\end{array}}^{20}{f}_{k}{\vartheta }({\xi }_{\hat{a}},{\xi }_{k})]\end{array}$$

where *f*_*k*_, 1 < *k* < 20 represents the frequency of pair function *ϑ* related to residue $\hat{a}$ with other nineteen amino acid residues and in case of non-occurrence, it is denoted by *f*_0_. Consider *ρ*_*l*,*m*_, it describes pair function *ϑ* for all amino acid residues with each other and while another pair function *ϑ*(*ξ*_*l*_,*ξ*_*m*_) in term of *ρ*_*l*,*m*_ is defined as *ϑ*(*ξ*_*l*_,*ξ*_*m*_) = *ρ*_*l*,*m*_; *l* = *m* = 1, 2, 3 … 20, shown in matrix (15). Subsequently, expression (16) is the complete representation for all possible pair factors regarding *X* and *Y* followed by (17), When pair *ϑ*(*ξ*_*l*_,*ξ*_*m*_) exists then *ρ*_*l*,*m*_ is established as 1 otherwise its assigned a zero entry. Furthermore, (16) admits to (15) with entries *ρ*_*l*,*m*_, *ρ*_*l*,*l*_ and *ρ*_*m*,*l*_ indicating lower triangular matrix for *X*. Consequently, diagonal entries represents the combination among similar residues and upper triangular matrix for *Y*.

15 $$(\begin{array}{ccccc}{\rho }_{\mathrm{1,1}} & {\rho }_{\mathrm{1,2}} & {\rho }_{\mathrm{1,3}} & \ldots  & {\rho }_{\mathrm{1,20}}\\ {\rho }_{\mathrm{2,1}} & {\rho }_{\mathrm{2,2}} & {\rho }_{\mathrm{2,3}} & \ldots  & {\rho }_{\mathrm{2,20}}\\ {\rho }_{\mathrm{3,1}} & {\rho }_{\mathrm{3,2}} & {\rho }_{\mathrm{3,3}} & \ldots  & {\rho }_{\mathrm{3,20}}\\ \vdots  & \vdots  & \vdots  & \ddots  & \vdots \\ {\rho }_{\mathrm{20,1}} & {\rho }_{\mathrm{20,2}} & {\rho }_{\mathrm{20,3}} & \ldots  & {\rho }_{\mathrm{20,20}}\end{array})$$

16 $$\begin{array}{c}(\begin{array}{ccccc}0 & 1 & 1 & \ldots  & 1\\ 1 & 0 & 1 & \ldots  & 1\\ 1 & 1 & 0 & \ldots  & 1\\ \vdots  & \vdots  & \vdots  & \ddots  & \vdots \\ 1 & 1 & 1 & \ldots  & 0\end{array})={M}_{i}={M}_{j}=\{{X}_{i}+{Y}_{i}\}=\{X+Y\}\\ \quad \quad \quad \quad \quad \quad \,\,=(\begin{array}{ccccc}0 & 0 & 0 & \ldots  & 0\\ 1 & 0 & 0 & \ldots  & 0\\ 1 & 1 & 0 & \ldots  & 0\\ \vdots  & \vdots  & \vdots  & \ddots  & \vdots \\ 1 & 1 & 1 & \ldots  & 0\end{array})+(\begin{array}{ccccc}0 & 1 & 1 & \ldots  & 1\\ 0 & 0 & 1 & \ldots  & 1\\ 0 & 0 & 0 & \ldots  & 1\\ \vdots  & \vdots  & \vdots  & \ddots  & \vdots \\ 0 & 0 & 0 & \ldots  & 0\end{array})\end{array}$$

where

17 $${\rho }_{l,m}=(\begin{array}{ll}1, & when\quad {\vartheta }({\xi }_{l},{\xi }_{m})\quad exists\quad for\quad both\quad l=m\quad or\quad l\ne m\\ 0, & otherwise\end{array}$$

Extension of (11) gives the idea for manipulating feature components for all twenty amino acid residues in matrix form, as given in (18).

18 $$\begin{array}{c}{{\rm{\Lambda }}}_{\hat{a}}+({{\rm{\Lambda }}}_{\hat{a}}-\mathrm{1)!}\,(\begin{array}{ccccc}1 & 0 & 0 & \ldots  & 0\\ 0 & 1 & 0 & \ldots  & 0\\ 0 & 0 & 1 & \ldots  & 0\\ \vdots  & \vdots  & \vdots  & \ddots  & \vdots \\ 0 & 0 & 0 & \ldots  & 1\end{array})+\frac{1}{38}[{(p-\mathrm{0)}}_{0}{M}_{0}+\sum _{q > p}\,{(q-p)}_{i}\,(\begin{array}{ccccc}0 & 1 & 1 & \ldots  & 1\\ 1 & 0 & 1 & \ldots  & 1\\ 1 & 1 & 0 & \ldots  & 1\\ \vdots  & \vdots  & \vdots  & \ddots  & \vdots \\ 1 & 1 & 1 & \ldots  & 0\end{array})\\ \,\,\,\,\,\,+\,{(N-r)}_{n}(\begin{array}{ccccc}0 & 1 & 1 & \ldots  & 1\\ 1 & 0 & 1 & \ldots  & 1\\ 1 & 1 & 0 & \ldots  & 1\\ \vdots  & \vdots  & \vdots  & \ddots  & \vdots \\ 1 & 1 & 1 & \ldots  & 0\end{array})]\end{array}$$

Using equation (14) in (12) contributes as a component of feature vector corresponding to residue $\hat{a}$ as given in equations (19) and (20).

19 $$\begin{array}{rcl}{{\rm{\Pi }}}_{\hat{a}} & = & {{\rm{\Lambda }}}_{\hat{a}}+({{\rm{\Lambda }}}_{\hat{a}}-\mathrm{1)!}\,{\vartheta }({\xi }_{\hat{a}},{\xi }_{\hat{a}})\\  &  & +\,\frac{1}{38}[{(p-\mathrm{0)}}_{0}{\{\sum _{\begin{array}{c}k\mathrm{=1}\\ k\ne \hat{a}\end{array}}^{20}{f}_{k}{\vartheta }({\xi }_{k},{\xi }_{\hat{a}})+\sum _{\begin{array}{c}k\mathrm{=1}\\ k\ne \hat{a}\end{array}}^{20}{f}_{k}{\vartheta }({\xi }_{\hat{a}},{\xi }_{k})\}}_{0}\\  &  & +\,\{{(q-p)}_{1}{\{\sum _{\begin{array}{c}k\mathrm{=1}\\ k\ne \hat{a}\end{array}}^{20}{f}_{k}{\vartheta }({\xi }_{k},{\xi }_{\hat{a}})+\sum _{\begin{array}{c}k\mathrm{=1}\\ k\ne \hat{a}\end{array}}^{20}{f}_{k}{\vartheta }({\xi }_{\hat{a}},{\xi }_{k})\}}_{1}\\  &  & +\,{(q-p)}_{2}{\{\sum _{\begin{array}{c}k\mathrm{=1}\\ k\ne \hat{a}\end{array}}^{20}{f}_{k}{\vartheta }({\xi }_{k},{\xi }_{\hat{a}})+\sum _{\begin{array}{c}k\mathrm{=1}\\ k\ne \hat{a}\end{array}}^{20}{f}_{k}{\vartheta }({\xi }_{\hat{a}},{\xi }_{k})\}}_{2}\\  &  & +\,{(q-p)}_{3}{\{\sum _{\begin{array}{c}k\mathrm{=1}\\ k\ne \hat{a}\end{array}}^{20}{f}_{k}{\vartheta }({\xi }_{k},{\xi }_{\hat{a}})+\sum _{\begin{array}{c}k\mathrm{=1}\\ k\ne \hat{a}\end{array}}^{20}{f}_{k}{\vartheta }({\xi }_{\hat{a}},{\xi }_{k})\}}_{3}\\  &  & +\,\cdots +{(q-p)}_{n-1}{\{\sum _{\begin{array}{c}k\mathrm{=1}\\ k\ne \hat{a}\end{array}}^{20}{f}_{k}{\vartheta }({\xi }_{k},{\xi }_{\hat{a}})+\sum _{\begin{array}{c}k\mathrm{=1}\\ k\ne \hat{a}\end{array}}^{20}{f}_{k}{\vartheta }({\xi }_{\hat{a}},{\xi }_{k})\}}_{n-1}\}\\  &  & +\,{(N-r)}_{n}{\{\sum _{\begin{array}{c}k\mathrm{=1}\\ k\ne \hat{a}\end{array}}^{20}{f}_{k}{\vartheta }({\xi }_{k},{\xi }_{\hat{a}})+\sum _{\begin{array}{c}k\mathrm{=1}\\ k\ne \hat{a}\end{array}}^{20}{f}_{k}{\vartheta }({\xi }_{\hat{a}},{\xi }_{k})\}}_{n}]\end{array}$$

Or

20 $$\begin{array}{rcl}{{\rm{\Pi }}}_{\hat{a}} & = & {{\rm{\Lambda }}}_{\hat{a}}+({{\rm{\Lambda }}}_{\hat{a}}-\mathrm{1)!}\,{\vartheta }({\xi }_{\hat{a}},{\xi }_{\hat{a}})\\  &  & +\,\frac{1}{38}[{(p-\mathrm{0)}}_{0}{\{\sum _{\begin{array}{c}k\mathrm{=1}\\ k\ne \hat{a}\end{array}}^{20}{f}_{k}{\vartheta }({\xi }_{k},{\xi }_{\hat{a}})+\sum _{\begin{array}{c}k\mathrm{=1}\\ k\ne \hat{a}\end{array}}^{20}{f}_{k}{\vartheta }({\xi }_{\hat{a}},{\xi }_{k})\}}_{0}\\  &  & +\,\sum _{q > p}\,{(q-p)}_{i}{\{\sum _{\begin{array}{c}k\mathrm{=1}\\ k\ne \hat{a}\end{array}}^{20}{f}_{k}{\vartheta }({\xi }_{k},{\xi }_{\hat{a}})+\sum _{\begin{array}{c}k\mathrm{=1}\\ k\ne \hat{a}\end{array}}^{20}{f}_{k}{\vartheta }({\xi }_{\hat{a}},{\xi }_{k})\}}_{i}\\  &  & +\,{(N-r)}_{n}{\{\sum _{\begin{array}{c}k\mathrm{=1}\\ k\ne \hat{a}\end{array}}^{20}{f}_{k}{\vartheta }({\xi }_{k},{\xi }_{\hat{a}})+\sum _{\begin{array}{c}k\mathrm{=1}\\ k\ne \hat{a}\end{array}}^{20}{f}_{k}{\vartheta }({\xi }_{\hat{a}},{\xi }_{k})\}}_{n}],\,i=1,2,3,\ldots ,n-1.\end{array}$$

**Figure 6:**
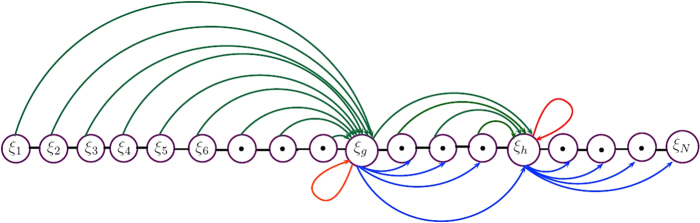
An illustration of the structural scheme of proposed technique.

To understand the structural scheme of proposed model consider *g*_*th*_ term of sequence given in equation (7), say, *ξ*_*g*_, which reflects the first alphabetical letter of amino acid residues say ‘A’. Notice its occurrences as well as corresponding positions in the sequence. *ξ*_*g*_ makes pair with its contiguous residues before and after the *g*_*th*_ residue in the terms *ϑ*(*ξ*_*k*_,*ξ*_*g*_) and *ϑ*(*ξ*_*g*_,*ξ*_*k*_) represented by green and blue curved lines and pairs *ξ*_*g*_ by itself represented by red loops (see Fig. 6). This process will be continued until next *ξ*_*h*_ occurs at *h*_*th*_ position such that *ξ*_*g*_ = *ξ*_*h*_ = *A*. Similarly same steps will be applied for *ξ*_*j*_. The feature component corresponding to residue “A” is interpreted in equation (21).

21 $$\begin{array}{rcl}{{\rm{\Pi }}}_{A} & = & {{\rm{\Lambda }}}_{A}+({{\rm{\Lambda }}}_{A}-\mathrm{1)!}\,{\vartheta }(A,A)\\  &  & +\,\frac{1}{38}[{({p}_{g}-\mathrm{0)}}_{0}{\{\sum _{\begin{array}{c}k\mathrm{=1}\\ k\ne A\end{array}}^{20}{f}_{k}{\vartheta }({\xi }_{k},A)+\sum _{\begin{array}{c}k\mathrm{=1}\\ k\ne A\end{array}}^{20}{f}_{k}{\vartheta }(A,{\xi }_{k})\}}_{0}\\  &  & +\,({q}_{h}-{p}_{g})\{\sum _{\begin{array}{c}k\mathrm{=1}\\ k\ne A\end{array}}^{20}{f}_{k}{\vartheta }({\xi }_{k},A)+\sum _{\begin{array}{c}k\mathrm{=1}\\ k\ne A\end{array}}^{20}{f}_{k}{\vartheta }(A,{\xi }_{k})\}\\  &  & +\,(N-{r}_{h})\{\sum _{\begin{array}{c}k\mathrm{=1}\\ k\ne A\end{array}}^{20}{f}_{k}{\vartheta }({\xi }_{k},A)+\sum _{\begin{array}{c}k\mathrm{=1}\\ k\ne A\end{array}}^{20}{f}_{k}{\vartheta }(A,{\xi }_{k})\}]\end{array}$$

where *k* = 1, 2, 3 …, 20 represents the ordinal values of twenty amino acid residues in alphabetical order and for more simplification assume that *ξ*_1_, *ξ*_2_, *ξ*_3_, …, *ξ*_20_ represents 20 amino acids in alphabetical order labeled as: A, C, D, E, F, G, H, I, K, L, M, N, P, Q, R, S, T, V, W and Y also *ξ*_21_ onwards the 20 residues cyclically repeats themselves then let Π_1_, Π_2_, Π_3_, …, Π_20_ be their corresponding feature components. The set of twenty feature components is given in equation (22).

22 $$\begin{array}{rcl}{{\rm{\Pi }}}_{1} & = & {{\rm{\Lambda }}}_{1}+({{\rm{\Lambda }}}_{1}-\mathrm{1)!}\,{\vartheta }({\xi }_{1},{\xi }_{1})\\  &  & +\,\frac{1}{38}[{(p-\mathrm{0)}}_{0}{\{\sum _{\begin{array}{c}k\mathrm{=1}\\ k\ne 1\end{array}}^{20}{f}_{k}{\vartheta }({\xi }_{k},{\xi }_{1})+\sum _{\begin{array}{c}k\mathrm{=1}\\ k\ne 1\end{array}}^{20}{f}_{k}{\vartheta }({\xi }_{1},{\xi }_{k})\}}_{0}\\  &  & +\,\sum _{q > p}\,{(q-p)}_{i}{\{\sum _{\begin{array}{c}k\mathrm{=1}\\ k\ne 1\end{array}}^{20}{f}_{k}{\vartheta }({\xi }_{k},{\xi }_{1})+\sum _{\begin{array}{c}k\mathrm{=1}\\ k\ne 1\end{array}}^{20}{f}_{k}{\vartheta }({\xi }_{1},{\xi }_{k})\}}_{i}\\  &  & +\,{(N-r)}_{n}{\{\sum _{\begin{array}{c}k\mathrm{=1}\\ k\ne 1\end{array}}^{20}{f}_{k}{\vartheta }({\xi }_{k},{\xi }_{1})+\sum _{\begin{array}{c}k\mathrm{=1}\\ k\ne 1\end{array}}^{20}{f}_{k}{\vartheta }({\xi }_{1},{\xi }_{k})\}}_{n}],\,i=1,2,3,\ldots ,n-1.\end{array}$$

$$\begin{array}{rcl}{{\rm{\Pi }}}_{2} & = & {{\rm{\Lambda }}}_{2}+({{\rm{\Lambda }}}_{2}-\mathrm{1)!}\,{\vartheta }({\xi }_{2},{\xi }_{2})\\  &  & +\,\frac{1}{38}[{(p-\mathrm{0)}}_{0}{\{\sum _{\begin{array}{c}k\mathrm{=1}\\ k\ne 2\end{array}}^{20}{f}_{k}{\vartheta }({\xi }_{k},{\xi }_{2})+\sum _{\begin{array}{c}k\mathrm{=1}\\ k\ne 2\end{array}}^{20}{f}_{k}{\vartheta }({\xi }_{2},{\xi }_{k})\}}_{0}\\  &  & +\,\sum _{q > p}\,{(q-p)}_{i}{\{\sum _{\begin{array}{c}k\mathrm{=1}\\ k\ne 2\end{array}}^{20}{f}_{k}{\vartheta }({\xi }_{k},{\xi }_{2})+\sum _{\begin{array}{c}k\mathrm{=1}\\ k\ne 2\end{array}}^{20}{f}_{k}{\vartheta }({\xi }_{2},{\xi }_{k})\}}_{i}\\  &  & +\,{(N-r)}_{n}\{\sum _{\begin{array}{c}k\mathrm{=1}\\ k\ne 2\end{array}}^{20}{f}_{k}{\vartheta }({\xi }_{k},{\xi }_{2})+\sum _{\begin{array}{c}k\mathrm{=1}\\ k\ne 2\end{array}}^{20}{f}_{k}{\vartheta }({\xi }_{2},{\xi }_{k})\}],\,i=1,2,3,\ldots ,n-1.\end{array}$$

$$\begin{array}{rcl}{{\rm{\Pi }}}_{3} & = & {{\rm{\Lambda }}}_{3}+({{\rm{\Lambda }}}_{3}-\mathrm{1)!}\,{\vartheta }({\xi }_{3},{\xi }_{3})\\  &  & +\,\frac{1}{38}[{(p-\mathrm{0)}}_{0}{\{\sum _{\begin{array}{c}k\mathrm{=1}\\ k\ne 3\end{array}}^{20}{f}_{k}{\vartheta }({\xi }_{k},{\xi }_{3})+\sum _{\begin{array}{c}k\mathrm{=1}\\ k\ne 3\end{array}}^{20}{f}_{k}{\vartheta }({\xi }_{3},{\xi }_{k})\}}_{0}\\  &  & +\,\sum _{q > p}\,{(q-p)}_{i}{\{\sum _{\begin{array}{c}k\mathrm{=1}\\ k\ne 3\end{array}}^{20}{f}_{k}{\vartheta }({\xi }_{k},{\xi }_{3})+\sum _{\begin{array}{c}k\mathrm{=1}\\ k\ne 3\end{array}}^{20}{f}_{k}{\vartheta }({\xi }_{3},{\xi }_{k})\}}_{i}\\  &  & +\,{(N-r)}_{n}{\{\sum _{\begin{array}{c}k\mathrm{=1}\\ k\ne 3\end{array}}^{20}{f}_{k}{\vartheta }({\xi }_{k},{\xi }_{3})+\sum _{\begin{array}{c}k\mathrm{=1}\\ k\ne 3\end{array}}^{20}{f}_{k}{\vartheta }({\xi }_{3},{\xi }_{k})\}}_{n}],\,i=1,2,3,\ldots ,n-1.\\  &  & \vdots \end{array}$$

$$\begin{array}{rcl}{{\rm{\Pi }}}_{20} & = & {{\rm{\Lambda }}}_{20}+({{\rm{\Lambda }}}_{20}-\mathrm{1)!}\,{\vartheta }({\xi }_{20},{\xi }_{20})\\  &  & +\,\frac{1}{38}[{(p-\mathrm{0)}}_{0}{\{\sum _{\begin{array}{c}k\mathrm{=1}\\ k\ne 20\end{array}}^{20}{f}_{k}{\vartheta }({\xi }_{k},{\xi }_{20})+\sum _{\begin{array}{c}k\mathrm{=1}\\ k\ne 20\end{array}}^{20}{f}_{k}{\vartheta }({\xi }_{20},{\xi }_{k})\}}_{0}\\  &  & +\,\sum _{q > p}\,{(q-p)}_{i}{\{\sum _{\begin{array}{c}k\mathrm{=1}\\ k\ne 20\end{array}}^{20}{f}_{k}{\vartheta }({\xi }_{k},{\xi }_{20})+\sum _{\begin{array}{c}k\mathrm{=1}\\ k\ne 20\end{array}}^{20}{f}_{k}{\vartheta }({\xi }_{20},{\xi }_{k})\}}_{i}\\  &  & +\,{(N-r)}_{n}{\{\sum _{\begin{array}{c}k\mathrm{=1}\\ k\ne 20\end{array}}^{20}{f}_{k}{\vartheta }({\xi }_{k},{\xi }_{20})+\sum _{\begin{array}{c}k\mathrm{=1}\\ k\ne 20\end{array}}^{20}{f}_{k}{\vartheta }({\xi }_{20},{\xi }_{k})\}}_{n}],\,i=1,2,3,\ldots ,n-1.\end{array}$$

The set of above twenty feature components are based on three properties of amino acids, namely hydrophobicity, hydrophilicity and side chain mass of amino acids. Each property associates a set of sixty components so collectively this admits 180 components manipulated by using equations (23) to (25), where *s* represents the number of attributes for amino acid residues in succinct representation, for *s* = 1, 2, 3 it corresponds to hydrophobicity, hydrophilicity and side chain mass of amino acids respectively.

23 $$\begin{array}{rcl}{\vartheta }({\xi }_{l},{\xi }_{m}) & = & \sqrt{{\Delta }_{s}^{\ast }{({\xi }_{l})}^{2}|{\Delta }_{s}^{\ast }({\xi }_{l})-{\Delta }_{s}^{\ast }({\xi }_{m}{)|}^{2}}\\  &  & +\,\frac{|({{\rm{\Delta }}}_{1}^{\ast }({\xi }_{l})-{\overline{{\rm{\Delta }}}}_{1}^{\ast }(\hat{a}))\,({{\rm{\Delta }}}_{2}^{\ast }({\xi }_{m})-{\overline{{\rm{\Delta }}}}_{2}^{\ast }(\hat{a})|}{\sqrt{{\sum }_{l=1}^{20}{({{\rm{\Delta }}}_{1}^{\ast }({\xi }_{l})-{\overline{{\rm{\Delta }}}}_{1}^{\ast }(\hat{a}))}^{2}\,{\sum }_{m=1}^{20}{{({\rm{\Delta }}}_{2}^{\ast }({\xi }_{m})-{\overline{{\rm{\Delta }}}}_{2}^{\ast }(\hat{a}))}^{2}}}\end{array}$$

24 $$\begin{array}{rcl}{\vartheta }({\xi }_{l},{\xi }_{m}) & = & \sqrt{{{\rm{\Delta }}}_{s}^{\ast }{({\xi }_{l})}^{2}|{{\rm{\Delta }}}_{s}^{\ast }({\xi }_{l})-{{\rm{\Delta }}}_{s}^{\ast }({\xi }_{m}{)|}^{2}}\\  &  & +\,\frac{|({{\rm{\Delta }}}_{1}^{\ast }({\xi }_{l})-{\overline{{\rm{\Delta }}}}_{1}^{\ast }(\hat{a}))\,({{\rm{\Delta }}}_{3}^{\ast }({\xi }_{m})-{\overline{{\rm{\Delta }}}}_{3}^{\ast }(\hat{a})|}{\sqrt{{\sum }_{l=1}^{20}{({{\rm{\Delta }}}_{1}^{\ast }({\xi }_{l})-{\overline{{\rm{\Delta }}}}_{1}^{\ast }(\hat{a}))}^{2}\,{\sum }_{m=1}^{20}{({{\rm{\Delta }}}_{3}^{\ast }({\xi }_{m})-{\overline{{\rm{\Delta }}}}_{3}^{\ast }(\hat{a}))}^{2}}}\end{array}$$

25 $$\begin{array}{rcl}{\vartheta }({\xi }_{l},{\xi }_{m}) & = & \sqrt{{{\rm{\Delta }}}_{s}^{\ast }{({\xi }_{l})}^{2}|{{\rm{\Delta }}}_{s}^{\ast }({\xi }_{l})-{{\rm{\Delta }}}_{s}^{\ast }({\xi }_{m}{)|}^{2}}\\  &  & +\,\frac{|({{\rm{\Delta }}}_{2}^{\ast }({\xi }_{l})-{\overline{{\rm{\Delta }}}}_{2}^{\ast }(\hat{a}))\,({{\rm{\Delta }}}_{3}^{\ast }({\xi }_{m})-{\overline{{\rm{\Delta }}}}_{3}^{\ast }(\hat{a})|}{\sqrt{{\sum }_{l=1}^{20}{({{\rm{\Delta }}}_{2}^{\ast }({\xi }_{l})-{\overline{{\rm{\Delta }}}}_{2}^{\ast }(\hat{a}))}^{2}\,{\sum }_{m=1}^{20}{({{\rm{\Delta }}}_{3}^{\ast }({\xi }_{m})-{\overline{{\rm{\Delta }}}}_{3}^{\ast }(\hat{a}))}^{2}}}\end{array}$$

where ${{\rm{\Delta }}}_{1}^{\ast }$, ${{\rm{\Delta }}}_{2}^{\ast }$, ${{\rm{\Delta }}}_{3}^{\ast }$ represents the normalized hydrophobicity, hydrophilicity and side-chain mass respectively. The values used in (23) to (25) are normalized with (26), and standardized with preferred range such that (−R, R), where R is the number in which $\hat{a}$ amino acids are being standardized. The hydrophobicity values are taken from Tanford C.^64^, hydrophilicity assesses are assumed from Hopp T. P., Woods K. R.^65^ and side-chain mass values are generally available in any biochemistry text book.

26 $$\begin{array}{rcl}{{\rm{\Delta }}}_{1}^{\ast }(\hat{a}) & = & [\frac{2R}{({{\rm{\Delta }}}_{{1}_{(max)}}-{{\rm{\Delta }}}_{{1}_{(min)}})}({{\rm{\Delta }}}_{1}(\hat{a})-{{\rm{\Delta }}}_{{1}_{(max)}})]+R\\ {{\rm{\Delta }}}_{2}^{\ast }(\hat{a}) & = & [\frac{2R}{({{\rm{\Delta }}}_{{2}_{(max)}}-{{\rm{\Delta }}}_{{2}_{(min)}})}({{\rm{\Delta }}}_{2}(\hat{a})-{{\rm{\Delta }}}_{{2}_{(max)}})]+R\\ {{\rm{\Delta }}}_{3}^{\ast }(\hat{a}) & = & [\frac{2R}{({{\rm{\Delta }}}_{{3}_{(max)}}-{{\rm{\Delta }}}_{{3}_{(min)}})}({{\rm{\Delta }}}_{3}(\hat{a})-{{\rm{\Delta }}}_{{3}_{(max)}})]+R\end{array}$$

Feature set is characterized with a vector having two hundred and twenty (220) elements, where the first sixty components based on the hydrophobic nature of amino acids, second sixty represents their hydrophilic nature, next sixty components contain information regarding side chain mass of 20 amino acids and the last forty elements reflect the positions as well as composition of individual amino acids residues. This novel predictor establishes outstanding outcomes towards recognition of roll down protein sequences. These extracted vectors obtained for training data are further used to train Neural Networks (NN) based classifier.

**Figure 7:**
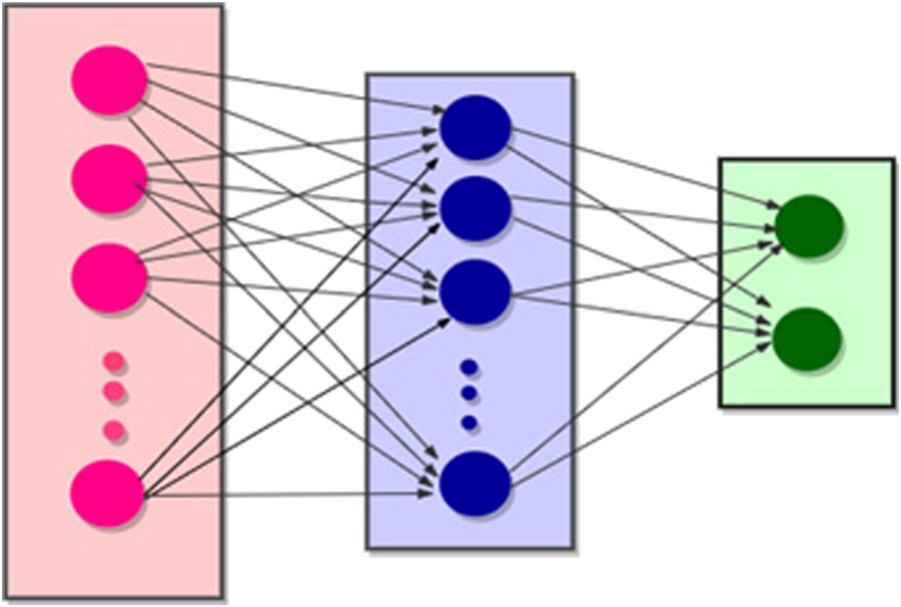
An illustration of the input, hidden and output layers of the neural network.

Neural networks are one of the most powerful techniques used to solve decision problems. They work by receiving labelled inputs and hence gain experience which help them to develop an opinion regarding arbitrary input for test purposes. After training process is completed the network seemingly behaves in a way that makes it capable to classify each given input within an acceptable degree of accuracy. During the learning process the network adjusts its weights such that the error is minimized which essentially translates into improved learning and increased accuracy^17^. A multilayer neural network was used to tackle this problem (see Fig. 7). The feature vector constructed for the prediction of signal peptides consists of 220 coefficients. Its connectionist architecture consists of 220 input layer neurons, 50 hidden layer neurons and two output neurons that discern among secretory and non-secretory poly-peptide chains. The training of the multilayered neural network is performed using back propagation method. In order to reduce the error and increase the prediction accuracy gradient descent technique was used along with an adaptive learning rate.

Respective output units are ultimately brought together through individual units of input, output representation^19^.

**Figure 8:**
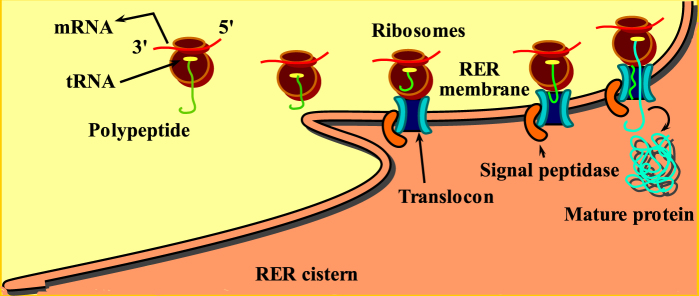
Signal peptidase is an enzyme that removes the signal of translocated primary proteins from the membrane to exhibit its mature form when they are substituted from a cytoplasmic position of synthesis to extracytoplasmic regions. Ultimately, these cleaved signal peptides are directed towards secretory tract.

Signal peptidase perform a momentous role in order to cleave signal sequence and the mature peptide from the nascent protein. Signal peptide is found in the vicinity of N-terminus site of protein sequence. Customarily, signal consists of 3–60 amino acid residues^8^. Translocon duct allows the passing of signal sequence transversely (see Fig. 8). Peptidases firstly confronts a nascent protein within endoplasmic reticulum (ER)^66^. Signal peptidase is also encountered in prokaryotes^67^ When protein builds appurtenance for mitochondria and chloroplasts.

A signal cleavage site for signal peptide is revealed by dividing concatenation into 3 parts, N-terminal part is basic positively charged and is labeled as the n-region, the central hydrophobic region is labeled as h-region and the C-terminal part describes the more polar site of the sequence and is represented as a c-region. Signal is encountered through the n-region to h-region by occupying positions −3 and −1. The application of “(−3, −1) -rule” proves very productive in identifying signal cleavage site directly. As discussed previously the dataset is divided in two categories, eukaryotes and prokaryotes (Gram-positive and Gram-negative) to predict the signal sequence and its cleavage site in both categories. Signal is mostly encountered embedded within the concatenation segregating the signal sequence and mature protein chain. The cleavage site of a secretory protein is determined by following these steps: First count the amino acid residues at each position in the sequence, formally, P($\hat{a}$, i), where P is the count factor for the occurrence of residue of type $\hat{a}$ at position *i*. Subsequently build Weight-matrices Q($\hat{a}$, i) by dividing all counts by their diverse expected abundance in proteins, primarily, $\langle P(\hat{a})\rangle$. Taking the natural logarithms of these entities for all sequences arrayed from their accepted sites of removed peptide chain between positions −1 and +1, t follows in equation (27).

27 $$Q(\hat{a},i)=ln(\frac{P(\hat{a},i)}{\langle P(\hat{a})\rangle })$$

Eukaryotic and prokaryotic Signal concatenation splitter follows the “(−3, −1) -rule”^10,23^. Statistical analysis shows that any residue out of Ala, Ser, Gly, Cys, Thr is placed at −1 location respect to the cleavage site while −3 site is occupied by Asp, Glu, Lys, Arg, Asn, Gln, (but not Phe, His, Tyr, Try), furthermore, there is no Pro residue between −3 to +1. Similarly, for Prokaryotic proteins same rule applies with a different set of residues. The −1 location is occupied by any of Ala, Gly, Ser, Thr whereas −3 is occupied by any of Ala, Gly, Leu, Ser, Thr, Val, also, −7 and −8 is mostly occupied by Leu or any other hydrophobic residue other than Val, Phe. In addition, it was also submitted that Proline (Pro) necessarily will be missed in the region −3 over +1.

**Figure 9:**
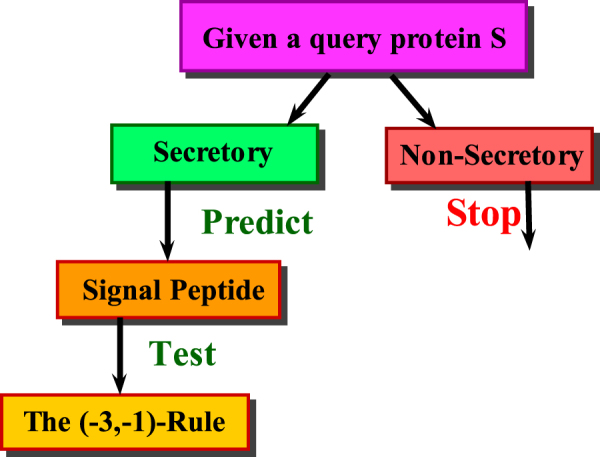
Pictorial representation shows how to predict the signal peptide and its cleavage site by means of proposed tool and the “(−3, −1)”-rule.

A scanning algorithm was developed to search the cleavage site pattern. It transcribed a weight matrix onto the polypeptide sequence. The residues bearing significance in identifying the cleavage site were assigned higher non-zero values while others were substituted by a zero value, hence the primary sequence was transformed into a vector. The algorithm worked by identifying a spike among the neighboring elements of the vector^10,23^. Systematic drawing reflects the overall procedure involved in predicting a signal peptide and determining its cleavage site (see Fig. 9).

## Electronic supplementary material

Supplementary Table S1

Supplementary Table S2
